# Fermented Food in Asthma and Respiratory Allergies—Chance or Failure?

**DOI:** 10.3390/nu14071420

**Published:** 2022-03-29

**Authors:** Anna Dębińska, Barbara Sozańska

**Affiliations:** Department and Clinic of Paediatrics, Allergology and Cardiology, Wrocław Medical University, ul. Chałubińskiego 2a, 50-368 Wrocław, Poland; barbara.sozanska@umw.edu.pl

**Keywords:** diet, fermented food, asthma, allergy, primary prevention

## Abstract

In the last few decades, a dramatic increase in the global prevalence of allergic diseases and asthma was observed. It was hypothesized that diet may be an important immunomodulatory factor influencing susceptibility to allergic diseases. Fermented food, a natural source of living microorganisms and bioactive compounds, has been demonstrated to possess health-promoting potentials and seems to be a promising strategy to reduce the risk of various immune-related diseases, such as allergic diseases and asthma. The exact mechanisms by which allergic diseases and asthma can be alleviated or prevented by fermented food are not well understood; however, its potential to exert an effect through modulating the immune response and influencing the gut microbiota has been recently studied. In this review, we provide the current knowledge on the role of diet, including fermented foods, in preventing or treating allergic diseases and asthma.

## 1. Introduction

The prevalence of allergic diseases and asthma has increased significantly in the last decades, especially in the western world [1]. This phenomenon has been attributed mainly to changes in environmental exposures and lifestyle. It is understood that a lack of natural stimulation of the immune system by the microbes from the external environment favors an allergic response. Our hygienic, modern lifestyle lacking sufficient contact with nature changed our immunity and reduced the diversity of our internal microbiome [2]. Moreover, a diet based on highly processed food, with high-fat and high-sugar products, can also promote inflammation and sensitization [3]. It is an urgent need to search for realistic ways to stop this allergic epidemic and to successfully implement the new methods of its prevention.

Presently in the western world, we observe the fashion for including natural ingredients in the diet, which increases the popularity of fermented food. However, fermented food has been a part of the human diet for centuries. In the past, fermentation was a method of natural preservation of meat, fish, vegetables, fruits, and dairy products. The benefits of a diet containing fermented milk were first described by the Nobel Prize winning microbiologist Ilya Metchnikoff over a century ago. He noticed that people who regularly consumed fermented yogurts regularly lived longer, and proposed that this may be connected with the beneficial action of lactobacilli [4]. In recent years, numerous studies have confirmed the health-promoting effects of fermented products. The metabolic and enzymatic activity of microorganisms involved in fermentation can exert anti-inflammatory and antioxidant action. Interestingly, it can also modulate the immune system and affect cytokine production and the composition and function of the gastrointestinal microbiota [5].

In this paper, we present the current knowledge on the role of fermented food in asthma and respiratory allergies, with special attention given to the possibilities of their application as a potential primary prevention mechanism for these diseases. We present the latest experimental and clinical research and discuss the mechanisms of action of different types of fermented food in allergic diseases and asthma. Treating and preventing asthma and respiratory allergies with a diet containing fermented products appears to be a promising strategy.

## 2. Types of Fermented Foods

Fermented foods are generally defined as foods and beverages created through desired microbial growth and enzymatic conversion of food components [6]. The wide range of food and microbial combinations provides a huge amount and variety of fermented foods that can be categorized using the several classifications based on the primary metabolites and microorganisms involved, the different food substrates, the methods of fermentation, and the primary source of fermentative microorganisms [7,8]. However, as stated by the above definition proposed by International Scientific Association for Probiotics and Prebiotics (ISAPP), food fermentation processes require the presence of microorganisms and their enzymes, as the activities of endogenous or exogenous enzymes alone are inadequate to consider the food as fermented [6,9]. The most essential and frequently used microorganisms in food fermentation processes are different species of bacteria, yeasts, and filamentous fungi [10,11]. Among bacteria, lactic acid bacteria (LAB), mostly *Lactobacillus*, *Lactococcus,* and *Tetragenococcus,* are widely present in many fermented foods, especially fermented milk and soy products [10,12]. Apart from LAB, certain species of acetic acid bacteria (AAB) are associated with fermentation processes of vinegar and kombucha or support meat and cheese fermentation, as in the cases of *Staphylococcus*, *Enterococcus*, *Brevibacterium,* and *Propionibacterium* [8,10,13]. Species of *Bacillus* are present in legume-based fermented foods, such as natto, miso, and soy sauce, made from soybeans [6,10]. Among thousands of species of fungi, the most commonly used are ethanol-fermenting yeasts (e.g., *Saccharomyces*) involved in the fermentation of wine and beer bread [10,11]. Different genera of molds, such as *Penicillium*, *Aspergillus,* and *Rhizopus,* are widely associated with dairy, meat, and soy product fermentation [6,10,11,13].

Microorganisms specify the type and outcomes of fermentation processes that can be categorized as alcoholic and anaerobic lactic acid fermentations, which occur in yoghurt, kimchi, and wine, or aerobic fermentations used by fungi in the processing of soy sauce and miso and by AAB in the production of vinegar and kombucha [10,11,14].

Fermentation can also be classified on the basis of the primary source of fermentative microorganisms [6,8]. Natural or spontaneous fermentation depends on the autochthonous microorganisms that naturally occur in the raw substrate or processing environment, as is the case in the production of various fermented soy products, fermented cereals, and fermented vegetables. This method is also used in the manufacturing process of wine, beer, and some fermented sausages [11,14,15]. The second type of fermentation, often referred to as “culture dependent”, is a technique using specific starter cultures that can be either natural or selected commercially. The starter culture approach is broadly applicable, and is used for preparation of dairy products, cheese, fermented sausages, and soy products such as kombucha, tempeh, and natto [11,15,16]. One of the methods used in culture-dependent fermentation is “backslopping”, in which a small quantity of the previously fermented food is inserted into raw food to start the new fermentation step. Backslopping is commonly applied in the production of sauerkraut, sourdough bread, and certain fermented soy products [7,11,15] (Table 1).

Of note, certain fermented products (e.g., beers, wines, bread) are commonly rid of the fermentation microorganisms before being distributed and may not comprise living microorganisms at the consuming stage. They should be categorized as “foods made by fermentation” [6,9]. Lastly, there are chemically derived versions of fermented food; for example, fruits and vegetables preserved by pickling processes, some salted or crude processed meats, and some chesses made by chemical acidification or chemically produced soy sauce and vinegar. These foods do not satisfy the definition of a fermented food [6,9] (Table 1).

## 3. Health-Promoting Components in Fermented Foods

Fermentation is an effective method of food processing which provides many valuable qualities, namely extended shelf-life, improvement of safety, attractive flavor and texture, enhanced nutritional and functional properties, and health benefits [7,8,17]. Epidemiological studies indicate that a diet with high consumption of fermented foods can influence human well-being, enhance health and quality of life, and reduce disease risk [3,18,19,20,21]. There are many components found in fermented foods that can exert favorable impacts on human health and disease (Table 2) [5,9].

Primarily, fermented foods have been found to contain living, functional microorganisms in notable quantities, ranging between at least 10^6^ and 10^9^ microbial cells per gram [16,22]. Study results indicated that intake of fermented foods significantly enlarges the number of microorganisms in the diet, even by up to 10,000-fold [21,22]. Functional activities of microorganisms in fermented foods include potential probiotic activity, enhancement of food safety and bio-availability of nutrients, production of antioxidant and antimicrobial compounds, synthesis of bioactive and nutritive compounds, and modulation of gut microbiota and the immune system [7,23].

Many species present in fermented foods are genetically similar to or share physiological traits with strains used as probiotics; hence, the health benefits of fermented foods may be due to probiotic activity [5,7,16]. Yogurt, kefir, and other fermented dairy products represent one of the most attractive sources of probiotic strains, mainly *Lactobacillus* spp. and *S. thermophiles*, as it has been shown that cultured dairy products consistently contained the highest levels of LAB [5,12,16,20,24]. However, probiotic strains such as *Lactobacillus plantarum* and *Lactobacillus rhamnosus* are also incorporated in oat-based fermented beverages that also contain the prebiotic fiber β–glucan [25]. Probiotic properties have been reported for *Lactobacillus plantarum* isolated from sauerkraut and kimchi [8,26,27,28]. African fermented cereal porridges and gruels made from millet, sorghum, and maize contain a large number of LAB (e.g., *Lactobacillus fermentum*) classified as probiotics [16,29]. It can therefore be assumed that ingestion of these fermented foods containing large numbers of microbes belonging to species with proven health-promoting properties could affect intestinal epithelium and the immune system in a comparable way to that described for probiotic strains [7]. Fermentation-associated microorganisms, despite their transient nature, can also alter gut microbiota diversity and function [30,31]. Interestingly, in the case of many fermented foods (e.g., yogurt, kefir, kimchi), daily consumption of around 100 g of those products would be sufficient to achieve the dose of 10^10^ microbial cells, which is suggested to be necessary to change the gut microbiota and supply potential health benefits [16,32].

**Table 2:** Health-promoting components in fermented foods.

Fermented Food Compounds	Health-Promoting Activity	References
**Microorganisms**	Probiotic activity	[16,20,24]
**Bacteriocin, nisin, organic acids**	Antimicrobial activity	[23]
**ACE-inhibitory peptides**	Anti-hypertensive activity	[33]
**γ** **-aminobutyric acid (GABA)**	Anti-hypertensive activity Antioxidant activity	[34]
**Folate (vitamin B9), B12, riboflavin,** **pyridoxine, nicotinamide** **Vitamin K**	Increase of vitamin content Antioxidant activity	[5,35]
**Conjugated linoleic acid (CLA)**	Antioxidant, anti-obesogenic, anti-carcinogenic, anti-atherosclerotic activity	[36,37,38]
**Phenolic compounds** **phenolic acids, flavonoids, saponins**	Immunomodulation, anti-hypertensive, antioxidant, anti-diabetic, anti-allergic activity	[39,40,41,42,43]
**Ferulic acid**	Anti-inflammatory activity Immunomodulation	[44,45]
**Cordycepin**	Antioxidant activity	[46]
**Phenolic, resveratrol**	Anti-inflammatory activity	[47,48]
**Ginsenoside**	Anti-inflammatory activity	[49,50,51]
**Isoflavones: genistein, daidzein, glycitein**	Antioxidant activity Anti-inflammatory activity Immunomodulation	[52,53,54,55]
**Aglycones, poly-gamma-glutamic acid (** **γ** **-PGA)**	Anti-inflammatory activity Immunomodulation	[56]

Additionally, microbial activity during food fermentation results in the improvement of food safety and nutritional composition. Numerous species of LAB derived from fermented vegetables and milk products produce antimicrobial end-products, such as organic acids, ethanol, bacteriocin, and nisin, and thereby diminish the risk of pathogen contamination [5,11,23,24,34]. Food fermentation can also result in the reduction or removal of toxic and antinutritive compounds. For example, fermentation may degrade the cyanogenic glycosides from bitter casava, remove the trypsin inhibitor in soybeans, or remove the phytic acid in cereals such as sorghum [6,7,23]. Sourdough fermentation can reduce immune-reactive proteins such as the amylase-trypsin inhibitor in wheat and fermentable oligosaccharides, disaccharides, monosaccharides, and polyols (FODMAPS) in wheat and rye bread [5]. The metabolic activity of microorganisms during fermentation may improve the bioavailability and tolerability of food components (e.g., lactose in dairy, fructans in wheat or raffinose in soybean and legumes) and may convert certain compounds to biologically active metabolites [23,34]. During the fermentation of plants and vegetables, LAB can convert phenolic compounds, increasing the bioavailability of flavonoids, saponins, tannins and other bioactive compounds [23,39].

Fermentation-related microorganisms have the ability to synthesize numerous metabolites and enhance the food with new nutrients and bioactive molecules that can exhibit either beneficial or harmful properties to human health [9]. In particular, organic acids (e.g., lactic acid and acetic acid), bioactive peptides (e.g., angiontensin-1-converting enzyme (ACE) inhibitors), phenolic compounds (e.g., phenolic acids, flavonoids), and exopolysaccharides are broadly distributed between both dairy and non-dairy fermented foods and have been shown to have health-promoting properties, such as immunomodulation, antioxidant, anti-hypertensive, anti-diabetic, and anti-allergic potential [17,33,34,57]. Amino acids and derivatives, mainly biogenic amines (e.g., beta-phenylethylamine and gamma aminobutyric acid (GABA)), are synthesized during fermentation and might serve as neurotransmitters and immunomodulators [9]. Another fermented dairy component, conjugated linoleic acid (CLA), is known for its health-promoting effects, including anti-obesogenic, anti-carcinogenic, anti-atherosclerotic and anti-inflammatory properties [36,57]. The fermentation of the diverse dietary fibers results in higher levels of several short-chain fatty acids (SCFA), especially butyrate, propionate, and acetate, which have immunostimulatory effects and significantly impact neuronal and metabolic functions [58]. The high antioxidant activity of fermented foods is related to the presence of phenolic compounds, flavonoids, GABA, CLA, folates, and bioactive peptides. Antioxidant activities have been observed in fermented milk, kimchi, fermented soybean foods, and fermented grain-based products [5]. The vitamin B complex including folic acid, riboflavin, pyridoxine, nicotinamide, and B12 is synthesized from various vitamin precursors by certain bacteria in dairy and plant-based fermented foods [5,6]. Vitamin K compounds are detected in high amounts in kimchi, fermented dairy such as kefir or matured cheeses, and fermented soybean foods such as natto [35].

Finally, according to observational and interventional studies, fermented foods can also modulate the gastrointestinal microbiota composition or function and redress the dysbiosis [59,60]. Among the fermented food compounds recognized to affect the composition of the gut microbiota are living microorganisms, microbial products and metabolites released as a results of fermentation (e.g., SCFA), and prebiotics (e.g., β-glucan) [5]. However, these effects may be transient and subject-specific, possibly dependent on differences in host physiology and the composition of the gut microflora [31].

Based on the knowledge of health-promoting components in fermented food and their potential ability to interplay with the immune system and the intestinal microbiota, the intake of fermented foods seems to be a promising strategy to reduce the risk of several disorders, including immune-related diseases, such as allergic diseases and asthma.

## 4. Potential Mechanisms of Action in Respiratory Allergic Diseases and Asthma

The exact mechanisms by which allergic diseases and asthma might be reduced or prevented by consuming fermented foods are uncertain. The most widely accepted theories are that fermented foods lead to anti-allergic effects either by modifying/influencing the gut microbiota or systemically modulating the immune response. Additionally, fermentation is an efficient approach for reducing or eliminating food allergenicity.

### 4.1. Immune Modulation

Immunomodulation applies to the concept that fermented foods can change the activity of both the local and systemic immune system, leading to upregulation or downregulation of the response to a successive challenge [24]. The immunomodulatory effect may result both from the increased production of immunoregulatory compounds and from direct influence on the cells of the immune system and epithelium [61,62]. The gastrointestinal tract appears to be the primary site where fermented foods interact with the immune system and exert their immunomodulatory, considering that approximately 70% of the entire human immune system is found in the gut [63]. Numerous mechanisms through which fermented foods exert their influence on the immune system have been proposed (Table 3).

Firstly, fermented food components might modify mucosal immunity and restore or enhance the barrier function of epithelium, either in the gut or lung. Indeed, fermentation-derived metabolites could increase the expression of tight junction proteins between epithelial cells and regulate the mitogen-activated protein kinases (MAPK) signaling pathway, thereby inhibiting the permeability of allergens via paracellular diffusion route [64,65,66,67]. Additionally, some fermented foods can hamper dendritic cells’ (DCs) maturation and reduce their migration effectivity and ability to activate T cell differentiation into allergic type Th2 cells [52,68,69]. Most importantly, it has been well-documented that fermented foods maintain a balance between the Th1 and Th2 response through upregulating the Th1 pathways while downregulating the Th2-related cytokine production [62]. In experimental studies, different fermented foods components and LAB were found to significantly stimulate the production of IFN-γ, TNF-α, IL-6, IL-12, and IL-1 in in vitro cell culture [71,72,73]. This upregulation of Th1 immune pathways could serve to regulate the overexpression of Th2-related immune responses [62]. Indeed, fermentation-associated microorganisms and bioactive molecules exhibited significant reduction in the IL-4, IL-5, and IL-13 concentration and Th2 cytokine-mediated eosinophilic airway inflammation, including reduction of IgE class switching (IL-4), eosinophils recruitment (IL-5), smooth muscle hyperreactivity, goblet cell hyperplasia and submucosal gland hyperplasia, and mucus hyperproduction (IL-13) [70,74,75,76,77]. Additionally, fermented foods have been shown to suppress the Th2-mediated allergic response by induction of CD4+CD25+ regulatory T-cells (Treg), which strongly downregulated Th2 cytokines production and inhibited the development and differentiation of Th2 cells [68,69,72,79]. The in vivo results suggest that LAB and other fermented food components also have the ability to attenuate IgE production [40,73,79,81]. The reduction of the serum total IgE and antigen-specific IgE in the immune study models using OVA-immunized mice may be related to upregulation of the proportion of CD4+CD25+ Treg cells and a decrease in maturation of B lymphocytes [72]. Another mechanism by which fermented foods may exert their anti-allergic effects relates to mast cells, which play a key role in allergic responses, as their degranulation results in the release of various mediators, such as histamine, prostaglandins, leukotrienes, and cytokines [84,85]. The experimental findings demonstrated the potency of fermented foods to decrease the expression of FcεR-related genes, thus suppressing IgE-mediated degranulation of mast cells [81,86]. Finally, murine experiments indicated that fermented food components may induce apoptosis in eosinophils, prevent their adhesion to endothelial cells, and reduce airway infiltration by eosinophils [41,53,77,78]. Regulation of these target mechanisms presented in Table 3 can alleviate allergic inflammation and reduce the symptoms of allergic diseases and asthma.

### 4.2. Modulation of Gut Microbiota

Diet is an important modifiable factor influencing the gut microbiota [88]. Fermented foods may modulate the gut microbiota composition and counteract proinflammatory effects of gut dysbiosis, which have been suggested to promote the development of allergic diseases [59,89,90,91]. It was demonstrated that fermented food components such as live microorganisms, their cell components, dietary fiber, and bioactive compounds (mainly polyphenols) modify the composition of gut microbiota by increasing the growth of beneficial genera, namely *Bifidobacterium*, *Lactobacillus,* and *Akkermansia,* and altering the ratio of *Firmicutes* to *Bacteroides* [58,59,60,92]. How this beneficial impact on gut microbiota influences local and systemic immunity and ultimately lung function remains unclear. Recent evidence suggested immune interactions between gut microbiota and the lungs, known as the gut–lung axis [93,94,95,96]. The crosstalk between the gut and lungs results from a shared development path, as both gastrointestinal and respiratory tracts develop from the same embryonic organ, the foregut, and retain some anatomical and physiological similarities [95,97]. The precise mechanisms by which the gut impacts the lung immune response are not fully understood; however, the proposed pathway is associated with the transfer of microbial cells or their products to mesenteric lymph nodes by ACP, where they induce the priming and maturation of T and B cells. After the activation T and B cells migrate through lymph and blood from the mesenteric lymph nodes to the lung epithelium and lung nodes, they infiltrate these sites and continue to stimulate other immune cells [93,95]. Another proposed pathway includes the systemic transmission of surviving bacteria, cell fragments, bacterial products, and metabolites (e.g., SCFA) from the enteric mucosa to the lung epithelium by lymph and blood circulation. After entering the pulmonary circulation, they can lead to activation of DC and macrophages and T cells differentiation [93,94]. SCFAs in particular are important immunomodulatory metabolites and act as a bridge between the microbiota and the immune system. Evidence has accumulated that SCFAs exert the anti-allergic effects by affecting the function of many cells, including epithelial, innate, and adaptive immune cells, which may also apply to the lungs [58,96,98]. Interestingly, fermented foods improve the production of SCFA and increase the level of circulating SCFA in addition to affecting gut microbial composition [67]. Based on the results, one can assume that beneficial effects of fermented foods on asthma and respiratory allergies might be, at least in part, related to the potential influence on the gut microbiota and the immune interactions of the gut–lung axis. However, further research has yet to be performed to understand the complex and interconnected axis between gut and lung.

## 5. Evidence from Experimental and Clinical Studies

To date, the various types of fermented foods have been extensively studied using in vitro experiments or animal models to support their immunomodulatory and anti-allergic effects. Clinical studies on fermented foods have also been conducted, although there are considerably fewer.

### 5.1. Fermented Dairy Products

Fermented dairy products such as yogurt, kefir, and cheese have received special attention because these products remain the main source of living microorganisms, mainly LAB, that are capable of modifying the immune response [12,16,20,24]. For example, probiotic fermented milk (PFM) containing *Lactobacillus bulgaricus*, *Streptococcus thermophilus,* and *Lactobacillus paracasei* strains was observed to mitigate respiratory allergy in ovoalbumin (OVA)-sensitised BALB/c adult mice [71]. The main immunomodulatory effect observed after the intake of PFM was manifested by the significant decrease in the specific IgE level in the serum and in the bronchoalveolar lavage fluid (BALF) and modulation of the Th1/Th2 balance by promoting a Th1 response associated with the increase in the IL-10, IFN-γ, and specific IgG2a level [71]. More recently, the protective effect of PFM on allergy was tested using 21-day-old BALB/c mice that were administered PFM for 10 days prior to induction of allergy. PFM stimulated development and activation of intestinal immune cells and controlled the response inducing IgG and favoring Th1-balance with increases in the IFN-γ and IL-10 within the mucosa of the respiratory system [99].

The kefir-derived LAB strains *Lb. kefiranofaciens* and *Lb. kefiri* have also been shown to exhibit anti-allergenic effects regarding Th1/Th2 balance in the favor of Th1 cells by induction of IFN-γ, TNF-α, IL-6, IL-12, and IL-1β in murine peritoneal macrophages [24,72]. Additionally, *Lb. kefiranofaciens* M1 isolated from kefir grains was found to reduce the levels of IL-5, while increasing the levels of IL-12, elevating the percentage of CD4+CD25+ Tregs, and inhibiting the IgE production in OVA-sensitized Th2-polarized mice [72]. A further study using the OVA-allergic asthma mice demonstrated that *Lb. kefiranofaciens* can prevent the development of allergen-induced sensitization by an increase of Treg activity, a suppression of Th2 (IL-4, IL-5, IL-13) and Th17 cytokines in splenocytes and BAL fluid, and an inhibition of specific IgE production in serum. It was also clearly demonstrated that *Lb. kefiranofaciens* is able to suppress the features of the asthmatic phenotype, including AHR to methacholine, airway inflammation, eosinophil infiltration in the lung, the hypersecretion of mucus, and goblet and smooth muscle cell hyperplasia in OVA-allergic asthma mice [78]. Kefir and its constituent strains, in particular *Lb. kefiranofaciens*, have also been shown to modulate the gut microbiota composition by stimulating the growth of *Lactobacillus*, *Lactococcus,* and *Bifidobacterium* and decreasing *Proteobacteria* and *Enterobacteriaceae* abundance, as revealed in several animal studies. Moreover, kefir consumption altered the *Firmicutes/Bacteroidetes* ratio which has been associated with maintaining homeostasis [100,101,102].

Recently, *Lactobacillus helveticus* isolated from fermented milk and some types of hard or semi-hard cheese were selected as an immunomodulatory effect strain. Experimental study in a murine pollen allergy model indicated that *Lactobacillus helveticus* LH2171 significantly diminished antigen-induced and total IgE levels in the blood. Additionally, *Lactobacillus helveticus* LH2171 inhibited the proliferation of immune cells and cytokine secretion in the submandibular lymph node and upregulated the expression of IL-10 and Foxp3, a marker of Tregs, in Peyer’s patches [73]. Makino et al. investigated the anti-allergic potential of *Lactobacillus helveticus* LH2171 using the antigen-stimulated murine naïve splenocytes in vitro and demonstrated the upregulation of IFN-γ and IL-10 secretion, with the suppressive effect of this strain on IL-4 and IL-13 production. Surprisingly, the impact on Treg cells differentiation as well as inhibition of systemic anaphylactic reaction was not observed. Further analysis using a murine model for pollen allergy revealed that *Lactobacillus helveticus* LH2171 suppresses pollen allergy symptoms, including face scratching and sneezing frequency. However, the alleviation of symptoms was not accompanied by inhibition of antigen-specific IgE production [103].

A randomized, double-blind, placebo-controlled study conducted in adult patients with mite or house dust allergy indicated that the daily consumption of drinkable yogurt containing *Lactobacillus helveticus* LH2171 strain for 12 weeks significantly improved ocular and nasal symptoms, including the number of sneezes. However, no significant differences were found in concentrations of Th1 and Th2 cytokine and total IgE or antigen-specific IgE levels in the serum [104]. More recently, another randomized clinical trial revealed that the regular daily ingestion of fermented milk containing *Lactobacillus helveticus* LH2171 for 16 weeks improved nasal discomfort and congestion in subjects with perennial allergy. The nasal and blood eosinophil counts and the eosinophil dynamics were markedly decreased in the *Lactobacillus helveticus* LH2171 group compared to the placebo group, suggesting that the lower degree of “stuffy nose” in this group was possibly due to suppression of eosinophils [105].

More recently, the preventive effect of six *Lactobacillus* species on asthma via gut microbiome modulation has been investigated in the murine house dust mite (HDM)-induced model of asthma. These findings indicated that *Lb. reuteri* isolated from fermented milk was the most effective, and modulated gut microbiota by increasing the abundance of Lactobacillus, Bifidobacterium, and Enterococcus. *L. reuteri* altered the gut microbial function toward increased SCFA, mainly butyrate production, and in turn decreased total IgE, inhibited Th2-associated proinflammatory cytokines (IL-5, IL-13), and reduced airway inflammation [106]. A further study by this group demonstrated that five *Lactobacillus casei* strains, broadly used in fermented dairy products, exhibited preventive effects on HDM-induced asthma in mice by modulating both the immune response and the gut microbiota composition. *Lb. casei* strains decreased the number of lymphocytes, eosinophils, and neutrophils and the levels of Th2 cytokines (IL-4, IL-5, IL-13, IL-9) and Th17 cytokine (IL-17A) as well as chemokine (such as eotaxin-1) in BALF. In addition, *Lb. casei* strains inhibited both HDM-specific IgG1 and total IgE levels in the serum. However, the results are not consistent for all strains, as it has been shown that *L. casei1* did not affect the production of inflammatory cytokines and *L. casei1* has no impact on immunoglobulin levels, while *L. casei3* exhibited the highest preventive effect. Furthermore, all five *L. casei* strains improved the diversity of gut microbiota mostly by promoting the growth of Firmicutes and increasing the SCFA (acetate and propionate) contents [74]. Taken together, modulating the gut microbiota via *Lactobacillus* species, especially *L. reuteri* and *L. casei3* supplementation, may become an effective approach to prevent asthma and other allergic diseases.

Dairy products are a rich source of conjugated linoleic acids (CLA), a class of unsaturated fatty acids, and their content can be significantly enriched by LAB fermentation [34,57]. In the murine asthma model, CLA was demonstrated to prevent experimentally induced airway inflammation and airway hyperresponsiveness with significantly reduced IgE production, less mucous plugging of segmental bronchi, and significantly reduced IL-5 levels and eosinophils counts in BALF [107]. Based on these encouraging results, a randomized, placebo-controlled study was conducted in patients with birch pollen allergy which indicated that CLA consumption before and during the pollen season could moderately attenuate the inflammatory response [108]. Furthermore, MacRedmond et al. reported that ingestion of CLA significantly improves lung functions and tolerance for strenuous exercise in overweight mild asthmatic patients. However, the inhibitory effect of CLA on systemic and airway inflammation has not been proven [37]. More recently, the pilot study was undertaken to assess the immunomodulatory and clinical effects of daily consumption of yogurt with CLA in children and adolescents with diagnosed asthma. The results indicated that CLA modestly suppressed the inflammatory response, decreasing the IFN-γ and IL-4 production in PBMC. However, no relevant improvement was observed with regards to lung functions, clinical symptoms, or specific IgE concentration [38]. These findings are in line with the previous trial in asthmatics showing that CLA ingestion did not inhibit the airway inflammation [109].

### 5.2. Fermented Plant Products

Fermentation of different plants leads to the synthesis of various bioactive compounds that are not present in raw products, including dietary fiber metabolites, phospholipids, isoflavones, phenolic acids, saponins, and phytic acids [5]. The immunomodulatory functions of fermented plants are attributable to these phytochemicals as well as live microorganisms [67].

#### 5.2.1. Fermented Medicinal Plants

Experimental studies provide growing evidence for the anti-allergic and anti-asthmatic activity of fermented products based on herbal components or extracts derived from medicinal plants or fungi. For example, well-known medicinal fungus *Cordyceps militaris* fermented with a *Pediococcus pentosaceus* strain demonstrated anti-allergic activity by suppressing the release of proinflammatory cytokines, inhibition of the signal transduction cascade accountable for type I hypersensitive allergic reaction in IgE/Ag-activated RBL-2H3 cells, and decreasing infiltration of immune cells and skin swelling in the in vivo BALB/c mice model [46]. These effects might be attributed to increased content of cordycepin, an antioxidant for which protective effects on OVA-induced allergic inflammation by enhancing Treg differentiation and inhibiting Th17-immune responses have been previously reported [110]. The anti-inflammatory and anti-asthmatic effectiveness was also confirmed for the fermented extract of medicinal plants consisting of *Ramulus mori*, *Salvia plebeia,* and *Anthriscus sylvestris* in the experimental study using cells from the human lung epithelium in vitro and an OVA/LPS-induced asthma mouse model in vivo. The administration of this extract significantly inhibited the secretion of proinflammatory cytokines (IL-4, IL-17), which in turn decreased IgE level and inflammatory cell infiltration in the BALF and hence alleviated lung tissue inflammation [79]. The anti-allergic activity of the extract is most likely due to rosmarinic acid and polyphenolic molecule oxyresveratrol, both of which are known to exert anti-inflammatory effects by suppressing the NF-κB signaling pathway [47,48]. Saponins are another plant-derived bioactive component responsible for the immunomodulatory effect of fermented foods, the content of which increases significantly after fermentation [9,23]. Indeed, saponin-enriched fermented extract of *Asparagus cochinchinensis* has been found to exhibit the dose-depended anti-asthmatic effects expressed through decreasing specific IgE level as well as reduction of immune cells infiltration, secretion of mucus, and respiratory epithelium remodeling in vivo in the OVA-challenged asthma mice model [40]. Recently, it was demonstrated that fermented *Platycodon grandiflorum* root extracts containing triterpenoid saponin platycodin D inhibited IL-17E cytokine production, and in turn the numbers of eosinophils and total cells BALF obtained from LPS/OVA-induced asthma mice, which indicates their effect on reducing airway inflammation [41].

Ginseng, a dried root of the *Panax spp.*, has been used around the world as the functional food and herbal medicine for treatment of allergic diseases including asthma and allergic rhinitis. Scientific research strongly supports the anti-allergic properties of ginseng extracts, which are additionally enforced by the fermentation [49]. It was clearly demonstrated that the fermentation of *Panax ginseng* extract reinforced its anti-allergic effects in vitro and in vivo. Ginseng fermented with *Lactobacillus plantarum* more strongly inhibited secretion of FcεRI-mediated β-hexosaminidase and IL-4 in mast cells and IgE-mediated passive cutaneous anaphylaxis in mice compared with unfermented extracts [81]. Lee et al. reported the anti-allergic effects of fermented red ginseng, which were associated with the regulation of Th1/Th2 balance, decreased gut permeability, and intestinal inflammation as well as subsequent suppression of IgE in OVA-sensitized mice [50]. Furthermore, the anti-allergic activity of water-extracted, ethanol-extracted, and bifidobacterial-fermented extracts of red ginseng have been evaluated in an allergic rhinitis in vivo mice model. Of tested extracts, fermented red ginseng most potently suppressed OVA-induced nasal allergy symptoms and decreased IgE levels by suppressing the infiltration of eosinophils and mast cells as well as the differentiation of Th2 cells with subsequent reduction of IL-4, IL-5, and IL-13 concentration in BALF and nasal fluid [51]. Less consistent results were obtained in the study on food allergic reaction, demonstrating that red ginseng extract did not affect the antigen-specific IgE levels in the spleen cells of the OVA-sensitized mice. However, the observed increase in the amount of CD8+ and IFN-γ-positive cells and IgA production in the small intestine suggested that ginseng might thus inhibit the allergic response to foods [111]. In addition, fermented red ginseng restored OVA-induced gut dysbiosis by increasing Bacteroidetes and Actinobacteria populations while reducing Firmicutes populations [50]. Ginsenosides and polysaccharides are mainly accountable for the anti-allergic effects of fermented ginseng extracts [49].

Double-blind, placebo-controlled study of the safety and efficacy of fermented red ginseng has confirmed the significant improvement in both nasal congestions symptoms and overall quality of life after a 4-week medication in patients with persistent allergic rhinitis. In addition, skin reactivity to perennial allergens was markedly decreased in treated patients, suggesting that fermented red ginseng can effectively suppress allergic inflammatory reactions [112].

#### 5.2.2. Fermented Cabbage Products

Sauerkraut, one of the most popular and oldest types of fermented cabbage, was considered a functional food with an immunomodulatory activity, as it is a unique source of vitamins, phenolic compounds, and in particular, LAB [8,67]. The immunomodulatory effect was found for *Lactococcus lactis* A17 isolated from fermented cabbage in OVA-sensitized BALB/c mice in vivo model and was related to the modulation of TLR4 expression and subsequent suppression of IL-4 cytokine and specific IgE production [113]. Moreover, *Lactobacillus plantarum* extracted from traditional Chinese sauerkraut Paocai inhibited allergen-induced infiltration of the inflammatory cells in the lung tissues of BALB/c mice in vivo via the Tregs-dependent mechanism. All tested strains induced the Th1-associated cytokines; however, only one strain (Lp) has been shown to be effective in inhibiting the Th2 cytokine IL-4 production, suggesting the strain-dependent effect. Surprisingly, the expected effects on the levels of specific IgE were not found [114]. Kimchi, the traditional Korean fermented vegetable dish, is another unique probiotic food containing a large quanity of LAB that remain alive after the fermentation process and can modulate the immune responses and gut microbiome composition. For example, *Lactobacillus plantarum* K-1 isolated from kimchi can mitigate allergic diseases, such as anaphylaxis, atopic dermatitis, and rhinitis, by suppressing the expression of TNF-α and IgE-switching cytokine IL-4 via inhibition of NF-κB and AP-1 signaling pathways [115]. *Lactobacillus sakei*, another probiotic strain extracted from kimchi, was observed to restore the skin epidermis, diminish the skin lesions, and reduce IgE level in in vivo mouse models of DNCB-induced atopic dermatitis. Oral administration of this strain decreased the number of CD4+CD25+ T cells and B cells and suppressed the Th2-assosiated cytokines by in vitro stimulation of tolerogenic DCs and further promotion of Treg differentiation [68,69]. In addition, *Lactobacillus sakei* modulated the structure of gut microbiota, decreasing AD-related gut bacteria (*Arthromitus* and *Ralstonia*) abundance while increasing the relative abundance of *Ruminococcus* that might be associated with Treg activation, and significantly reduced symptoms in atopic dermatitis mice [68].

#### 5.2.3. Fermented Grain Products

LAB-fermented germinated brown rice is another plant food which looks promising in terms of the treatment of IgE-dependent allergic diseases, as it contains a high level of ferulic acid and protocatechuic acid, both known to have anti-allergic activities [82]. In addition, the concentration of these phenolic compounds was enhanced in course of LAB fermentation [44]. Results from the OVA-induced pulmonary allergy murine models indicated that ferulic acid and protocatechuic acid inhibited the epithelial derived cytokines (TSLP, IL-25, IL-33), restored Th1/Th2 imbalance by modulating DCs function, reduced serum level of the OVA-specific IgE, and decreased the key features of pulmonary allergy, such as airway hyperreactivity, eosinophilic inflammation, thickness of respiratory epithelium, hypersecretion of mucus, airway infiltration by inflammatory cells, and smooth muscle cell proliferation [45,70,75]. More recently, Dhong and Park demonstrated that germinated brown rice fermented with the *Pediococcus pentosaceus* exhibit a suppressive impact on IgE/Ag-mediated type I hypersensitivity reactions through inhibition of mast cell degranulation, reduction of proinflammatory cytokine expression, and negative regulation of the FcεRI-mediated signaling pathway [82].

#### 5.2.4. Fermented Fruits Products

Fermented fruits are a rich source of diverse micronutrients, dietary fiber, and phytochemicals such as anthocyanins, flavanols, resveratrol, and phenolic acids that have been found to present multiple health-promoting benefits [5,67]. A recent study evaluated the immunomodulatory effects of the traditional multi-fruit beverage containing a special composition of five fruits (kiwi, guava, papaya, pineapple. and grape) that are fermented by lactic and acetic acid bacteria in combination with *Saccharomyces cerevisiae.* In the in vivo study model, oral intake of the fermented multi-fruit beverage was demonstrated to change the Th1/Th2 balance by enhancement of Th1-type immune response with subsequent attenuation of Th2-related cytokine IL-4 and IL-5 secretion in OVA-sensitized BALB/c mice [76]. The immunomodulatory properties of fruits can be assigned to the phytochemicals, in particular anthocyanins and polyphenols; however, the active components of the fermented multi-fruit beverage have not yet been identified [67,76]. Berries are among the richest source of phenolic compounds, which have a greatly increased content and bioavailability after the fermentation process [42]. Evidence exists that fermented berries may exert health benefits including anti-allergic effects through the modification of gut microbiota [42]. Berry polyphenols affect gut microbiota, leading generally to an increase in beneficial genera, therefore suggesting a prebiotic-like activity of fermented berries or their phenolic compounds. Moreover, they increase the production of SCFA and improve mucosal immunity [43,67]. Recently, Cheng et al. demonstrated using an in vitro model that *Lactobacillus casei*-fermented blueberry pomace revealed potential gut-improving functions through suppressing the growth of *Escherichia coli* and *Enterococcus* and altering the ratio of *Firmicutes* and *Bacteroidetes* as well as the stimulation of *Bifidobacterium, Ruminococcus, Lactobacillus*, and *Akkermansia* genera. In addition, fermented blueberries increased the production of SCFA, especially acetic, butyric, and lactic acids [116]. These findings were further confirmed in the in vivo study investigating the effect of berry polyphenols supplementation on intestinal microbiota in mice recieving a high-fat diet with *L.casei*-fermented blueberry pomace [117].

Harima-Mizusawa et al. demonstrated the beneficial effect of citrus juice fermented with *Lactobacillus plantarum* YIT 0132 against allergic symptoms in an animal model of Japanese cedar pollinosis, which is the most common type of allergic rhinitis in Japan. In addition, the daily intake of citrus juice fermented with *Lactobacillus plantarum* alleviates symptoms and enhances quality of life in patients with Japanese cedar pollinosis during the natural pollen season [118]. Further double-blind, placebo-controlled trials in patients with perennial allergic rhinitis showed a significant reduction in the nasal symptoms score and stuffy nose score in subjects consuming *L.plantarum*-fermented juice. Furthermore, these benefits were associated with the immunomodulating activities of *Lactobacillus plantarum,* as the reduction of Th2 cells, total IgE, and eosinophil cationic protein level was observed [87].

#### 5.2.5. Fermented Soy Products

Numerous fermented soybean foods from different parts of Asia, namely tempeh, natto, miso, douche, and soy sauce, have long been consumed due to their well-known health benefits [8,119]. Evidence has shown that anti-allergic effects of fermented soybean products may be attributable to isoflavones, the major physiologically active substance in soybeans, the amounts of which increase significantly during the fermentation process [80,120]. Isoflavones, such as genistein, daidzein, and glycitein, have been shown to protect against allergic inflammation reactions and modulate the immune response through suppressing DC maturation and subsequent Th2 response as well as inhibiting IgE signaling and mast cell degranulation [52,54,55]. Genistein has also been revealed as a potential inhibitor of allergic responses by inhibition of FcεRI expression on human mast cells and blocking FcεRI-IgE interaction [86]. The fermented soy product ImmuBalance, made from defatted soybeans with *Aspergillus oryzae* and lactic acid bacteria (*Pediococcus parvulus* and *Enterococcus faecium*), is an excellent source of isoflavones and has been shown to suppress peanut hypersensitivity on a murine model of peanut allergy as evidenced by a reduction of Th2 cytokines (IL-4, IL-5 and IL-13) and peanut-specific IgE production [83]. Recently, it has been shown that intake of the fermented soy product ImmuBalance suppressed eosinophilic airway inflammation while reducing the density of infiltrating eosinophil and mucus accumulation in a murine model of OVA-induced asthma. Moreover, supplementation with ImmuBalance decreased the concentration of Th2 cytokine in BALF as well as the serum level of OVA-specific IgE [53]. The authors hypothesized that the anti-asthmatic effect of ImmuBalance would be attributed to increasing concentrations of SCFAs and influences on gut microbiota, as the modulatory effect of ImmunoBalance on gut homeostasis has been previously reported [121].

Cheonggukjang (fermented soybean paste), a traditional Korean fermented dish also called natto, tempeh, and douchi in other Asian regions, has recently emerged as a functional food with immunomodulation properties [80]. Cheonggukjang is produced from boiled soybeans rice straw, usually by fermentation with *Lactobacillus spp*., *Bacillus* strains (*B. subtilis* or *B. licheniformis*), *Enterococcus faecium,* and *Leuconostoc spp.* [56]. The anti-allergic effect of cheonggukjang on atopic dermatitis was demonstrated by amelioration of typical features of an allergic skin reaction, including decreased thickness of dermis and mast cells infiltration [84]. Later study on an in vivo atopic dermatitis mice model revealed that cheonggukjang markedly reduced the clinical scores of atopic dermatitis and epidermal thickness and suppressed mast cells infiltration, and degranulation, scratching behavior, and expression of IgE, IL-4, and IL-31 were observed. The results suggested that cheonggukjang inhibits IL-4 and itch-related IL-31 cytokine production by suppressing the activation of NF-κB and MAPK pathways [85]. Additionally, the therapeutic effect of cheonggukjang on airway inflammation has been investigated on a murine model of ovalbumin (OVA)-induced asthma. The results indicated that cheonggukjang downregulated the number of eosinophils and monocytes infiltrating the bronchial mucosa and suppressed the features of airway remodeling such as subepithelial fibrosis, goblet cell hyperplasia, and underproduction of mucus in mice with OVA-induced asthma [77]. These effects were attributed to isoflavone aglycones, poly-gamma-glutamic acid (γ-PGA), and oligosaccharides, the active compounds of cheonggukjang with known immunomodulatory activities [56]. Tempeh, the Indonesian type of fermented soybean paste, was demonstrated to have the ability to modulate gut microbiota composition due to high content of dietary fibers. In Sprague–Dawley rats, the supplementation with tempeh increased stool *Bacteroidetes*, *Clostridium leptum*, and *Bacteroides fragilis* and altered or decreased the ratio of *Firmicutes/Bacteroidetes* [122]. Moreover, the study using an in vitro simulator model of the digestive tract revealed that fermented soybean and bean tempeh exert an impact on human microbiota by stimulating the growth of *Bifidobacterium*, *Lactobacillus*, *Escherichia coli,* and *Enterococcus* species [123].

Questionnaire-based studies have reported that ingestion of foods with increased content of soy genistein might be correlated with better clinical control of asthma symptoms and improvement of lung function in adult patients [124,125]. However, a randomized clinical trial did not prove the therapeutic effect of soy compounds on poorly controlled asthma in adults and adolescents. The supplementation with soy isoflavones did not improve the lung function parameters or the control of asthma determined by symptoms severity and the number of exacerbation as well as the airway and systemic inflammation markers such as exhaled nitric oxide [126].

Recently, the pea (Pisum sativum), belonging to legumes, has received special attention, as it offers an opportunity to replace animal protein with a healthier, economically viable, and environmentally friendly source of protein. Pea seeds contain high amounts of essential amino acids and proteins, dietary fiber, minerals, vitamins, and phytochemicals that are related to their health beneficial properties [127]. Experimental studies have revealed that supplementation with fermented plant extract containing pea proteins might modulate gut microbiota and exert immunomodulatory effects [128,129,130]. Despite its nutritive value and health benefits, the consumption of pea remains restricted due to its unwanted sensory qualities, low solubility, and the content of non-nutritive compounds [131]. In several recently published studies, lactic acid fermentation has been shown to reduce undesirable aroma and taste, eliminate antinutritional ingredients, and improve the protein digestibility and bioavailability, increasing the nutritional value of pea [132,133,134,135]. Highly promising results were obtained for the fermentation by a co-culture of LAB and yeasts as well as the combination of fermentation and enzymatic hydrolysis [136,137]. In addition, it has been found that fermentation can decrease the allergenicity of pea proteins by changing allergenic epitopes [137,138]. Importantly, the usefulness of fermentation as an effective method of reducing the allergenicity has also been confirmed for other types of food, as reviewed by Pi et al. [139].

When summarizing the evidence from the studies reviewed here, it seems rational to conclude that the consumption of fermented foods can play a promising role in treatment or even prevention of asthma and respiratory allergies. The vast majority of published studies provide data supporting the positive anti-inflammatory, anti-allergic, and immunomodulatory potential of different fermented foods [49,61,62,67]. There are only limited studies where this beneficial impact has not been confirmed [37,74,103,104,105,111,114]. However, it should be emphasized that these observations come mainly from in vitro or animal studies, while clinical studies are scarce, and their results remain more inconsistent [37,38,104,105,107,108,124,126]. Naturally, we must consider some hazards for health associated with fermented foods resulting from the risk of the presence of microbial pathogens and microbial metabolites such as mycotoxins, salt, ethyl carbamate, acrolein, and biogenic amines [34,140]. In particular, fermented products containing high levels of histamine and tyramine can, in some circumstances, induce an allergy-like syndrome which closely imitates food allergy symptoms including diarrhea, respiratory distress, rhino-conjunctivitis, urticaria, pruritus, and headache [141]. On the other hand, certain fermented foods (e.g., dairy products, soy-based products, cereal-based products, lupins, peas, ginseng) can be a source of food allergen proteins that may cause an immune reaction in allergic patients [142]. It was demonstrated that fermentation efficiently destroys the allergenic proteins and thus markedly reduces the immunoreactivity of fermented foods; however, this might not be sufficient for complete elimination of remaining allergenicity in some products [141,142]. Therefore, further studies are still needed to conclude the hypoallergenic properties of fermented foods as well as their anti-allergic effectiveness.

## 6. Evidence from Epidemiological Studies

Evidence from epidemiological studies supports the hypothesis that dietary habits affecting the intestinal microflora may contribute to reducing the risk for developing allergies. One example is an anthroposophic lifestyle, characterized by high intake of fermented vegetables and biodynamic food, restrictive use of antibiotics, incomplete immunization, and prolonged breastfeeding [143]. In one cross-sectional study, frequent consumption of fermented vegetables, a characteristic of the anthroposophic lifestyle, was correlated with lower prevalence of allergic diseases and positive responses to objective tests of atopy in children [144]. The lower prevalence of current rhinoconjunctivitis symptoms, atopic eczema symptoms, and atopic sensitization was also reported among children attending Steiner schools leading an anthroposophic lifestyle in the PARSIFAL study [145]. Similar findings have also been found in the prospective birth cohort Assessment of Lifestyle and Allergic Disease During Infancy (ALADDIN), which demonstrated that leading an anthroposophic lifestyle was associated with a decreased prevalence of food allergen sensitization and episodes of wheeze reported by parents, but not eczema [146]. The above-mentioned studies, however promising, do not allow to determine one specific factor responsible for the preventive effect of the anthroposophic way of life. Consumption of fermented foods is one of the features of this lifestyle; therefore, it cannot be ruled out that an allergy-protective effect is probably related to complex interaction between various lifestyle factors and environmental exposures. However, another epidemiological study revealed that eating fermented food and buying food directly from farms reduced the risk of childhood allergies [147]. More recently, the South African Food Allergy (SAFFA) study identified the consumption of fermented milk (amasi) as a protective factor for asthma and atopic eczema [148]. In support of this, the authors from former studies further reported that specific anthroposophic lifestyle features influenced the composition of the gut microbiota in children with increased abundance and diversity of lactobacilli and a higher accumulation of acetic acids [149,150]. Furthermore, results from the ALADDIN birth cohort study indicated that six-month-old infants growing up in anthroposophic families had a significantly increased abundance of Bifidobacterium and reduced abundances of Bacteroides and Veillonella [151]. Fermented food intake may also reflect, at least in part, farming lifestyle, a factor with protective effects against asthma and allergic diseases that have consistently been demonstrated [152,153].

Data from the large Korea National Health and Nutrition Examination Survey (KNHANES) study conducted to assess the association between dietary factors and asthma in adults demonstrated a significant inverse correlation between kimchi consumption and the prevalence of adult asthma. It has been observed that patients with asthma reported a lower consumption of kimchi and fish compared to those without asthma [154]. In the next phase of the KNHANES study, using data from the 2011 and 2012, kimchi consumption was inversely correlated with the prevalence of rhinitis and atopic dermatitis in adults [155,156]. Interestingly, the prevalence of asthma, rhinitis, and atopic dermatitis decreased with increasing kimchi consumption. In particular, relative to group, which had the lowest kimchi intake (<1 serving; <40 g) subjects consuming ≥3 servings per day (≥120 g) had a 34% lower presence of asthma, a 19% lower presence of rhinitis, and a 32% lower presence of atopic dermatitis. Based on these results, it can be deduced that consuming the adequate amount of kimchi may reduce the risk of developing allergic diseases, including asthma [154,155,156].

When summarizing the results from epidemiological studies, it is important to highlight the potential limitation of this research method. Questionnaire-based assessment of exposure creates the possibility of recall or selection bias, as well as the risk of misunderstanding the question about the type of exposure. Furthermore, the correlations between outcomes and exposure found in the observational studies, even if biologically plausible, do not necessarily imply a causal relationship and require further analysis before making detailed conclusions.

## 7. Conclusions

Fermented foods have been considered as an essential element of the human diet for thousands of years, enriching it with nutritional compounds. Presently, the consumption of fermented foods is gaining increasing interest as an important dietary method for improving human health. Their excellent biological activities, in particular anti-inflammatory and immunomodulatory properties, are associated with high content of live microorganisms, phytochemicals, and bioactive compounds. Following the growing evidence from epidemiological studies, the Metchnikoff’s original theory can be extrapolated to the possibility that fermented foods could represent a promising strategy of treatment and prevention of asthma and respiratory allergies. As reviewed, evidence from in vitro experiments and animal trials reported in the literature thus far has shown that fermented foods could exert effects against allergic airway inflammation, possibly through modulating the local and systemic immune response and modifying the gut microbiota. Although the concept of the food–gut axis, and further, the gut–lung axis has been the subject of intense study in recent years, detailed mechanistic studies are still required to obtain insight on the association between diet, in particular fermented foods, and gut and lung microbiota composition and their impact on the immune response. Furthermore, most of our knowledge in this field is based solely on experimental studies, while there are only limited clinical evidence. Therefore, in the future, there is a critical need for preventive and treatment-oriented, well-designed, randomized, controlled clinical trials and broad population studies to provide support to the evidence found on the effectiveness of fermented food on asthma and respiratory allergies. These studies should focus on well-defined disease phenotypes, diverse immune-related parameters, the gut–lung microbiome composition, and analysis by metagenomics and metabolomics. However, designing and repeating studies on fermented foods may be a challenge due to the high variability of microbial cultures and ingredients present even in the same types of fermented foods. Nevertheless, it has been suggested that this effort is important, as the benefits of whole fermented foods are bigger than the sum of their individual components. Such efforts are particularly needed, as diet is one of the most modifiable and readily available interventions for every person and may represent a safe, non-invasive, non-pharmacological addition to disease management. Meanwhile, fermented foods remain a promising strategy for the treatment and prevention of asthma and respiratory allergies that could be included into dietary guidelines in the future.

## Figures and Tables

**Table 1:** Description of fermented foods based on [6,7,8,9,10,11].

Category	Type of Food	Substrate	Source of Organisms
**Fermented foods** “Contains live and active culture” live microorganisms present	Yogurt, kefir	Animal milk	Starte culture
	Cheese, sour cream	Animal milk	Starter culture, backslopping
	Miso	Soybean	Starter culture, spontaneous
	Natto, tempeh, douchi	Soybean	Starter culture, backslopping
	Sauerkraut, kimchi	Cabbage, green onion, hot pepper, ginger	Spontaneous
	Kombucha	Brewed black or gren tea	Starter culture (SCOBY)
	Boza, busher, mahewu, other fermented cereals	Cereals, maize, sorghum, millet	Spontaneous
	Sausage, peperoni, salami	Pork, beef	Backslopping, starter culture, spontaneous
**Fermented foods** “Foods made by fermentation” live microorganisms absent	Sourdough bread	Bread made from longer ferment	Spontaneous or backslopping
	Soy sauce	Soybean, wheat	Starter culture, spontaneous
	Vinegar	Grape, apple, malt, rie, fruits	Spontaneous, starter culture
	Wine	Grape	Spontaneous, starter culture
	Roasted coffee and chocolate beans	Coffee beans, cocoa beans	Spontaneous, starter culture
	Beer	Malted barley, water, hops	Blackslopping, starer culture
**Not fermented foods** chemically derived version of fermented food	Chemically leavened bread		
	Fresh sausage		
	Pickled vegetables		
	Chemically produced soy sauce and vinegar		
	Salted meats and fishes		
	Cheese made by chemical acidification		

**Table 3:** Proposed mechanisms of immunomodulatory action of fermented foods.

Target	Mechanism of Action	References
**The epithelium barrier** **(intestinal, lung)**	Enhancement the epithelial barrier function Inhibition of allergen penetration Increasing the expression of tight junction protein	[64,65,66,67]
**Dendritic cells (DCs)**	Reduction of DC maturation, migration and activation	[52,54,68,69,70]
**Th1 immune response**	Increasing the production of Th1 cytokine: INF-γ, TNF-α, IL-1, IL-6, IL-10, IL-12	[24,71,72,73]
**Th2 immune response**	Decreasing the production of Th2 cytokine: IL-4, IL-5, IL-13	[51,70,74,75,76,77]
**Regulatory T-cells (Treg)**	Induction of CD4+CD25+ Treg	[68,69,72,78,79,80]
**B cells and Immunoglobulins**	Decreasing in maturation of B lymphocytes Reduction of IgE isotype switching Reduction of serum total IgE and antigen specific IgE	[40,73,79,81,82,83]
**Mast cells**	Decreasing the expression of FcεR-related genes Inhibition of mast cells degranulation Suppression of histamine prostaglandins, leukotrienes and cytokines release	[81,82,84,85,86]
**Eosinophils**	Induction of apoptosis in eosinophils Inhibition of eosinophils migration Suppression of eosinophilic inflammation	[41,51,53,77,78,87]
**Airways**	Reduction of smooth muscle hyperreactivity, Reduction of goblet cell hyperplasia and mucus production Reduction of inflammatory cells (lymphocytes, eosinophils, neutrophils) in BALF Reduction of airway remodeling	[40,45,70,74,75,76,77,78,87]

## Data Availability

Not applicable.
